# Synergistic Neuroprotection in Tauopathic Mice via Green-Synthesized Silver Nanoparticles Co-delivering Methylene Blue and *Moringa oleifera*

**DOI:** 10.1007/s12035-025-05534-9

**Published:** 2025-12-28

**Authors:** Yasmin Elbermawy, Sara EL-Desouky, Reem K. Arafa, Sara Elfarrash, Ahmad Bassiouny

**Affiliations:** 1 Euro-Mediterranean Master of Neuroscience and Biotechnology (EMN-Online), Faculty of Science, Alexandria University, NeuroscienceAlexandria, Egypt; 2 Medical Experimental Research Center (MERC), Faculty of Medicine, Mansoura University, Mansoura, 35516 Daqahlia Egypt; 3 Drug Design and Discovery Lab, Zewail City of Science, Technology and Innovation, Helmy Institute of Medical Sciences, Giza, 12578 Egypt; 4 Biomedical Sciences Program, Zewail City of Science, Technology and Innovation, University of Science and Technology, Giza, 12578 Egypt; 5 Department of Medical Physiology, Faculty of Medicine, Mansoura University, Mansoura, 35516 Daqahlia Egypt; 6 Physiological Sciences Department- MBBS Program, Fakeeh College for Medical Sciences, 21461 Jeddah, Saudi Arabia; 7 Department of Biochemistry and Molecular Biology, Sealy Center for Molecular Medicine, University of Texas Medical Branch, Galveston,, TX, 77555 USA; 8 Department of Biochemistry, Alexandria University, Alexandria, 21511 Egypt; 9 Department of Otolaryngology, Louisiana State University Health Science Center, New Orleans, LA, 70112 USA

**Keywords:** Alzheimer’s Disease, Tauopathy, Methylene Blue, *Moringa Oleifera*, Nanotechnology-based Therapy, Neuroinflammation

## Abstract

Alzheimer’s disease (AD) is a progressive neurodegenerative disorder with limited therapeutic options.Most current treatments target only a single pathogenic pathway. We developed an innovative green-synthesized silver nanoparticle formulation for the co-delivery of methylene blue (MB), a tau aggregation inhibitor, and *Moringa oleifera* (MO) extract, an antioxidant and anti-inflammatory agent. Silver nanoparticles act as multifunctional carriers, improving drug stability and brain delivery, yielding the combined formulation MOMB-Ag-NPs. MOMB-Ag-NPs were synthesized and characterized using ultraviolet–visible spectroscopy (UV–Vis), transmission electron microscopy (TEM), scanning electron microscopy (SEM), and energy-dispersive X-ray (EDX) analysis. Homozygous P301S tau transgenic mice were assigned to four groups: saline (0.9%, i.p.), MB (4 mg/kg/day, i.p.), MO (260 mg/kg/day, oral), or MOMB-Ag-NPs (4 mg/kg/day MB equivalent, i.p.) for 60 days. In vitro GSK-3β inhibition assays and molecular docking analyses assessed mechanistic interactions. Neuroprotective efficacy was evaluated through survival, behavioral tests, immunohistochemistry, ELISA, and Western blotting. MOMB-Ag-NPs displayed spherical morphology (10–25 nm), high stability, and efficient MB encapsulation (EE 54.7%, DL 93.5%). Both MO and MB inhibited GSK-3β in vitro (IC50 = 9.41 and 65.77 µg/mL), corroborated by molecular docking. In vivo, MOMB-Ag-NPs significantly improved locomotor activity, and cognitive performance. Treated mice showed reduced astrogliosis, decreased pro-inflammatory cytokines (TNF-α, IL-6), enhanced autophagy (LC3β), increased antioxidant defenses (SOD), and differential modulation of the AKT/GSK-3β pathway. This study provides novel evidence that a green-synthesized MB and MO nanoformulation exerts synergistic neuroprotective effects in tauopathy mice, highlighting the translational promise of multitarget strategies for AD treatment.

**Supplementary Information:**

The online version contains supplementary material available at 10.1007/s12035-025-05534-9.

## Introduction

Alzheimer’s disease (AD) is a progressive neurodegenerative disorder and the leading cause of dementia [1]. Its multifactorial pathology involves progressive cognitive decline and neuropathological hallmarks including extracellular β-amyloid (Aβ) plaques and intracellular neurofibrillary tangles formed by hyperphosphorylated tau protein [2]. Many studies have revealed that tau pathology and chronic neuroinflammation are potentially more closely associated with cognitive decline than the presence of Aβ plaques [3]. Tauopathies encompass a group of disorders characterized by abnormal tau aggregation, including hyperphosphorylation and neurofibrillary tangle formation [4]. These hallmark lesions are accompanied by oxidative stress, mitochondrial dysfunction, and chronic neuroinflammation, ultimately driving synaptic and neuronal loss [5]. Despite being a major global health challenge, current treatment options remain limited in efficacy like symptomatic relief [6], and monotherapies, and most approved drugs focus on single targets, such as cholinergic deficits or Aβ accumulation yielding modest clinical benefits and raising safety concerns [7, 8]. These limitations highlight a critical gap: the multifactorial nature of AD cannot be effectively addressed by monotherapies, underscoring the urgent need for multi-targeted strategies capable of simultaneously modulating tau pathology, neuroinflammation, and oxidative stress [9]. In this context, our study investigated a multitarget therapeutic approach designed to address these interconnected pathogenic mechanisms.

Different targets were suggested among central molecular targets for AD therapy. Among prominent candidates is glycogen synthase kinase-3β (GSK-3β) which is a serine/threonine kinase regulating glucose metabolism, Wnt/β-catenin signaling, and neuronal survival [10]. Aberrant activation in AD promotes tau hyperphosphorylation, amyloidogenic APP processing, and neuroinflammation [11]. Consequently, GSK-3β inhibition represents a promising multitarget strategy [12]. Assessing GSK-3β inhibition in vitro [13], and in vivo [14]provides mechanistic validation that the bioactivity of both MB and MO-derived flavonoids, directly links the formulation to the therapeutic goal of developing a disease-modifying intervention.

In pursuit of such interventions, compounds such as methylene blue (MB) and *Moringa oleifera* (MO) have shown increasing interest. MB, a phenothiazine dye, has been reported previously to inhibit tau aggregation by oxidizing cysteine residues (Ser202/Thr205) [15], disrupting β-sheet structures [16], and promoting clearance [17]. While the anti-tau effect of MB demonstrated some conflicts, different mechanisms were suggested like supporting mitochondrial function via redox cycling, reducing oxidative stress, and modulating autophagy and inflammation [18, 19]. *Moringa oleifera* (MO), a flavonoid-rich medicinal plant, exerts antioxidant, anti-inflammatory, and antiapoptotic effects [20]. Different studies have suggested a potential neuroprotective effect of MO through quercetin that neutralizes ROS and enhances endogenous antioxidant enzymes through Nrf2/ARE activation [21], NF-κB signaling, lowering proinflammatory cytokines [21, 22], and shifting apoptosis toward survival via Bcl-2 upregulation and Bax/cytochrome c/caspase-3 suppression[22–25]. Myricetin further inhibits GSK-3β and promotes autophagy, reinforcing MO as a multitarget agent in AD therapy [26].

Despite their potential, both MB and MO have demonstrated pharmacokinetic limitations in different studies, including low brain bioavailability and potential toxicity at high doses [27, 28]. Our preliminary in vivo studies demonstrated that co-administration of unformulated MB and MO resulted in systemic toxicity and mortality, underscoring the need for an optimized delivery platform to enhance efficacy while minimizing adverse effects.

Building on recent advances in nanotechnology, we present an innovative nanotherapeutic strategy that utilizes a green-synthesized silver nanoparticle formulation (MOMB-Ag-NPs) for co-delivery of MB and MO. This dual-loaded platform enables controlled and sustained release at lower doses, improves blood–brain barrier penetration, and minimizes systemic toxicity [29]. Silver nanoparticles were specifically selected for their intrinsic antioxidant, anti-inflammatory, and neuroprotective properties, complementing the therapeutic objectives in AD, These properties make AgNPs an attractive nanocarrier system, while green synthesis ensures low toxicity and high encapsulation efficiency [30].

In the current study, we developed and evaluated a novel dual-loaded green-synthesized silver nanoparticle formulation (MOMB-Ag-NPs) for the co-delivery of MB and MO extract, with the goal of enhancing therapeutic efficacy while reducing systemic toxicity. To our knowledge, this is the first study to employ silver nanoparticles as dual nanocarriers for both MB and MO in the context of AD. The efficacy of the nanoformulation was assessed in P301S transgenic mice, a widely used tauopathy model that closely mimics tau hyperphosphorylation and neurofibrillary tangle formation in AD. MB and MO monotherapies were included as pharmacological comparators to determine the added value of the co-formulation. This approach offers a cost-effective [31], targeted, and minimally invasive means of delivering therapeutics, promoting localized action and enhanced efficacy.

## Materials and Methods

### Chemicals and Reagents

Silver nitrate (AgNO₃; Sigma-Aldrich, St. Louis, MO, USA; Cat. No. 209139, Lot# SLBV2452) was used as the precursor salt for nanoparticle synthesis. Methylene blue (MB; CDH, New Delhi, India; Cat. No. 034048, Lot# MB2023A) wasused for nanoformulation and dissolved in 0.9% saline to prepare stock solutions. Aqueous *Moringa oleifera* leaf extract was provided by the National Research Centre (Cairo, Egypt), prepared from authenticated dried leaves (Voucher No. NRC-MO-2022; Lot# MOE-0622). Phosphate-buffered saline (PBS, pH 7.4; Thermo Fisher Scientific, USA; Cat. No. 10010023, Lot# 2390402) was used for sample preparation. For kinase assays, a GSK-3β Kinase Enzyme System (BPS Bioscience, San Diego, CA, USA; Cat. No. 79700Z, Lot# 23C03) and Kinase-Glo® reagent (Promega, Madison, WI, USA; Cat. No. V6711, Lot# 0000582972) were employed.

### Preparation of Aqueous *Moringa oleifera* Extract

**Fig. 1:**
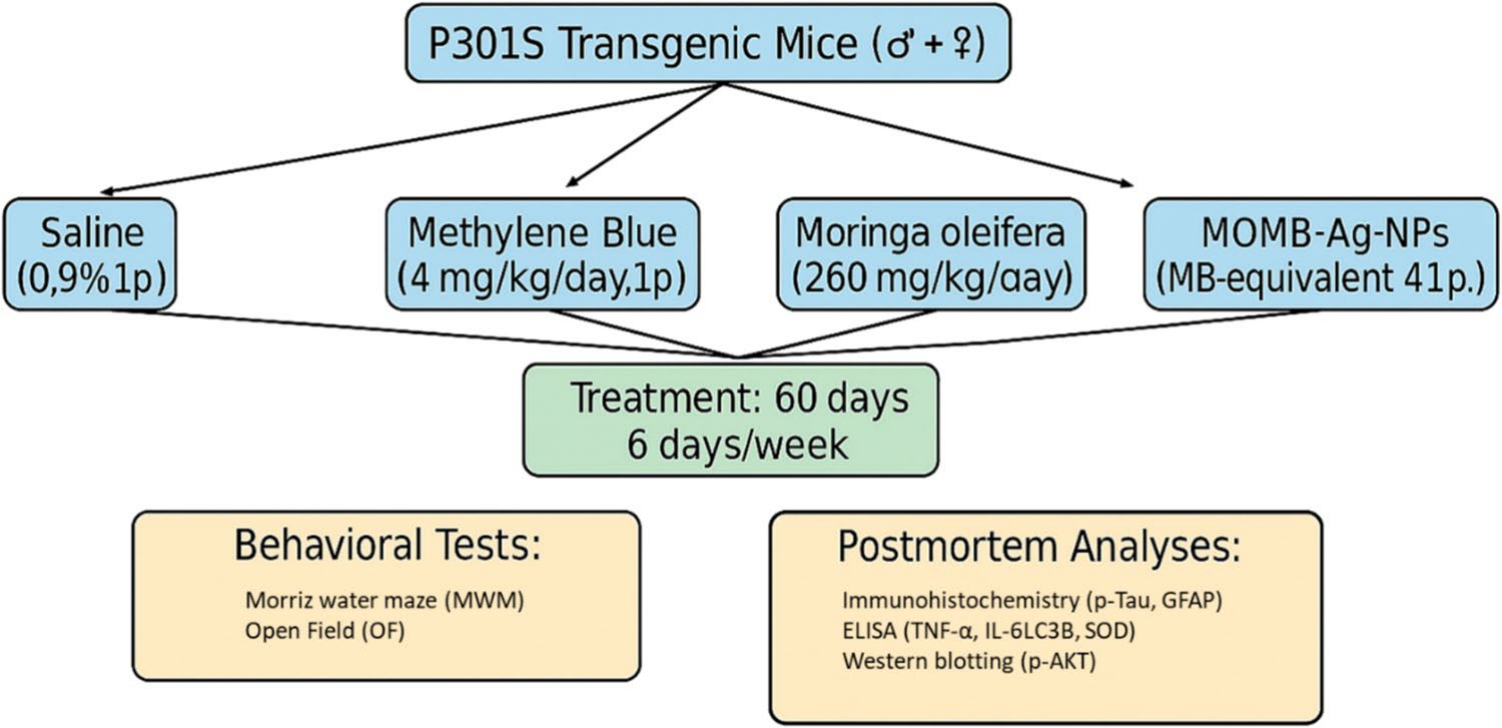
Schematic representation of experimental groups and treatment regimen

Dried *Moringa oleifera* leaves were finely ground into powder using a laboratory grinder to enhance extraction efficiency. The powder was extracted by maceration at a ratio of 1:1 (w/v) and incubated at 55 °C overnight to facilitate the extraction of the soluble constituents. The extract was filtered through muslin cloth, yielding a 100% aqueous extract. The obtained extract was transferred into Erlenmeyer flasks, sealed with cotton plugs, and heated at 50 °C for 15 min to minimize microbial contamination. The filtrate was subsequently concentrated under reduced pressure using a rotary evaporator (Büchi B-100) until the volume was reduced. The concentrated extract was cooled and stored until experimental use (Fig. 1) [32].

#### Phytochemical Profiling of *Moringa oleifera* Extract

Phytochemical characterization and targeted flavonoid identification were carried out as follows. The total phenolic content (TPC) was determined using the Folin–Ciocalteu assay and expressed as µg gallic acid equivalents per mL (µg GAE/mL), following the method described by (Attard 2013). The total flavonoid content (TFC) was assessed using the aluminum chloride colorimetric method and expressed as µg rutin equivalents per mL (µg RE/mL), according to (Kiranmai, Kumar, et al. 2011). Antioxidant capacity was evaluated using both 2,2-diphenyl-1-picrylhydrazyl** (**DPPH) and 2,2′-azino-bis(3-ethylbenzothiazoline-6-sulfonic acid) (ABTS) radical scavenging assays, and results were expressed as Trolox equivalents. Chromatographic phytochemical profiling was conducted to identifyconstituents of *Moringa oleifera* extract, which were analyzed by HPLC–PDA using a reverse-phase C18 column. PDA spectra were recorded across 200–400 nm. Major flavonoids and phenolic acids were identified by comparison of retention times and PDA spectral profiles with authenticated reference standards, including myricetin, apigenin-7-glycoside, and chlorogenic acid.

### Ag-Nanoparticle Preparation and Characterization

**Fig. 2:**
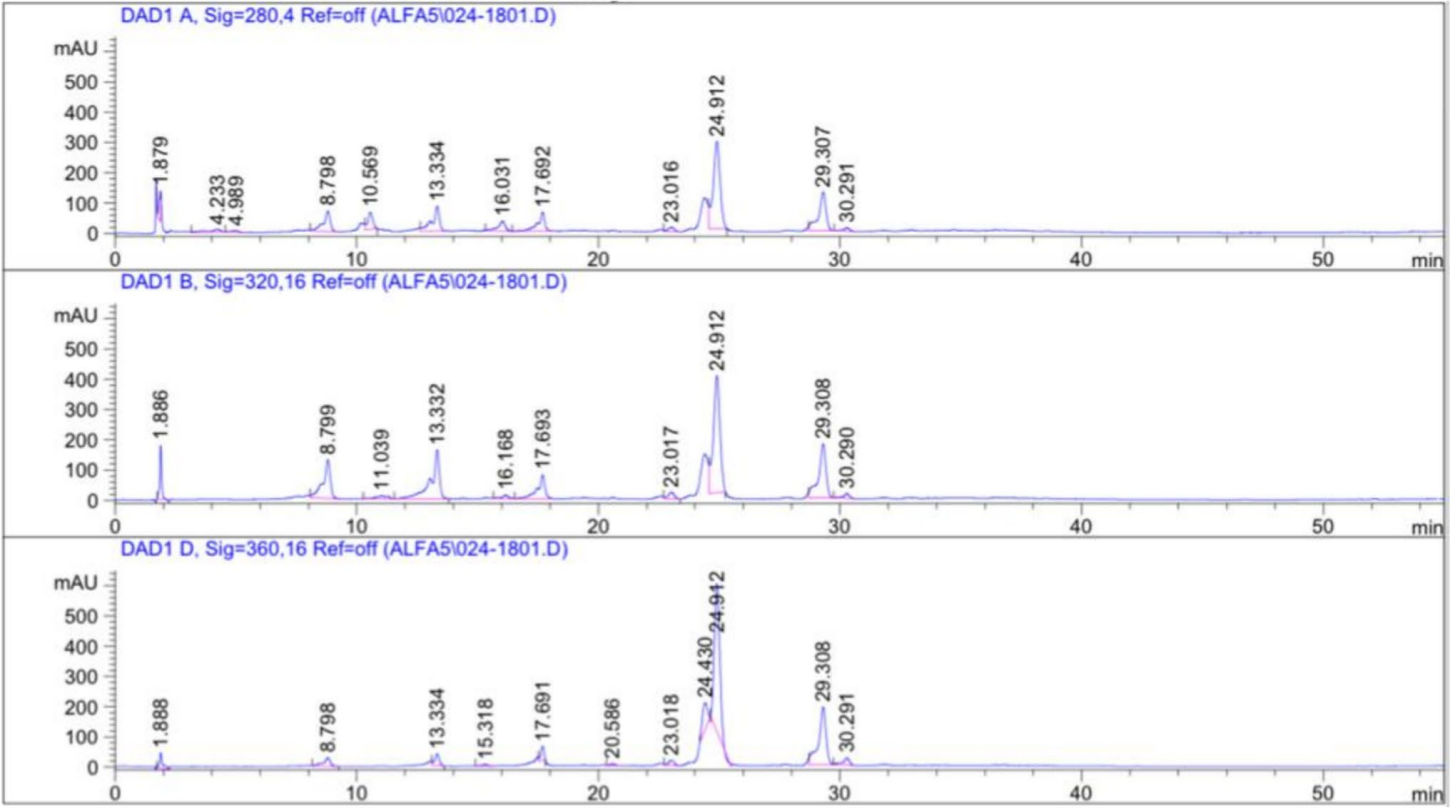
HPLC–PDA chromatograms of *Moringa oleifera* aqueous extract recorded at 280, 320, and 360 nm. Distinct peaks corresponding to phenolic and flavonoid constituents were observed, with major compounds identified as myricetin (rt = 24.9 min, 94.664 µg/mL), apigenin-7-glycoside (rt = 29.2 min, 55.960 µg/mL), and chlorogenic acid (rt = 13.2 min, 28.432 µg/mL), alongside minor phenolics such as rosmarinic acid, p-hydroxybenzoic acid, vanillic acid, and gallic acid. The chromatographic profile highlights myricetin as a principal flavonoid in the extract

The biosynthesis of MOMB-Ag-NPs via MO-Ag-NPs was performed at the Center for Microbiology and Phage Therapy (CMP), Zewail City of Science and Technology, following a previously described method with some modifications [33, 34]. Briefly, 1 mg of AgNO₃ was added to 1 mL of *Moringa oleifera* (MO) extract dissolved in 49 mL of distilled water, resulting in a final volume of 50 mL. The mixture was heated on a hot plate at 85 °C for 2 h with continuous stirring (400 rpm) and exposure to light.The appearance of a light-brown color indicated the formation of MO-stabilized silver nanoparticles (MO-Ag-NPs). Subsequently, 15.9 mg of methylene blue (MB; MW 319.85 g/mol) was added to the MO-Ag-NPs suspension, where it acted as both a catalytic and functionalizing agent, generating the final MOMB-Ag-NPs, and was maintained under the same conditions for an additional 2 h. The visible color change to deep blue yielded the final MB-coated MO-Ag-NPs (MOMB-Ag-NPs) (Fig. 2). The nanoparticles were collected by centrifugation at 12,000 × g for 15 min, washed with distilled water, and dried at 80 °C to obtain the final powder. The nanoparticles were finally re-dissolved in deionized water for in vivo administration.

#### Physicochemical Characterization of Nanoparticles

The physicochemical properties of the MOMB-Ag-NPs were characterized using multiple complementary techniques, with *Moringa oleifera* (MO) extract serving as the main reducing and stabilizing agent, which significantly influenced particle shape and size.

##### UV–Visible Spectroscopy

Ultraviolet analyzed optical properties–Vis spectroscopy (Shimadzu UV-1800, Japan)in the 330–800 nm range. The absorbance spectra of MOextract and silver nitrate solution were recorded without dilution after 2 h of reaction to monitor the reduction process. In contrast, the spectra of MOMB-Ag-NPs were obtained using appropriately diluted samples (1:10,000). A characteristic absorbance peak confirmed nanoparticle formation, the successful reduction of Ag⁺ ions, and subsequent functionalization with MB.

##### Transmission Electron Microscopy (TEM), Scanning Electron Microscopy (SEM) and Energy-Dispersive X-ray Spectroscopy (EDX)

TEM (JEOL JEM-2100, Japan, 200 kV) was used to visualize the two-dimensional structure and assess the size distribution of the biosynthesized nanoparticles.Morphological evaluation was performed using SEM (JEOL JCM-7000 NeoScope). Elemental composition was confirmed by EDX analysis, which detected silver, oxygen, and organic elements, verifying successful nanoparticle synthesis and surface coating by MO and MB.

##### Encapsulation Efficiency and Drug Loading

After synthesis, MB-loaded silver nanoparticles were separated from free drug by centrifugation (12,000 × g, 15 min). The concentration of unencapsulated MB in the supernatant was quantified by UV–Vis spectroscopy at 664 nm, using a calibration curve generated by serial dilutions of MB in distilled water. Encapsulation efficiency (EE%) and drug loading (DL%) were calculated as follows:

$$EE\%=\left(C_{total}-C_{free}\right)\backslash C_{total}\times100$$

$$DL\%=\left(C_{total}-C_{free}\right)\backslash W_{nanoparticle}\times100$$ [35] 

where *C*_total_ is the initial amount of MB used for formulation (15.9 mg), *C*_free_ is the amount of MB detected in the supernatant after centrifugation (7.2 mg), and *W*_nanoparticle_ is the weight of the freeze-dried nanoparticles (9.3 mg).

### In Vitro Studies

#### GSK-3β Enzyme Inhibition Assay

The in vitro enzyme inhibition capacity of MO and MB was determined via a GSK-3βAssay Kit obtained from BPS Bioscience, USA (catalogue #79700Z). This kit is designed to measure GSK-3β activity for screening and profiling applications and uses Kinase-Glo® (Promega) as a detection reagent. The IC_50_ values of MO and MB were calculated from the dose–response curves obtained upon assessing the inhibitory effects of serial dilutions of the tested materials (0.01, 0.1, 1, 10, and 100 µg/mL). The experiments were conducted following the supplier’s standard procedure [36].

### In Silico Studies

#### Molecular Docking Study

To further understand the molecular mechanism of action of the tested agents, a molecular docking study of the most abundant polyphenolic flavonoid components of MO, myricetin, and MB was conducted on GSK-3β (PDB ID: 7B6F). MOE v.2019.01 was deployed for this study, and default parameters were used for docking employing the Amber10:EHT forcefield. Triangular Matcher and London dG scoring were utilized for placement, whereas Rigid Receptor and GBVI/WSA dG scoring were used for refinement.

### Animals and Housing

Homozygous P301S transgenic mice overexpressing human tau on a C57BL/6 background were obtained and maintained at the Mansoura Experimental Research Center (MERC), Mansoura University, Egypt [37–40]. Mice were 16–17 weeks old, and were randomly divided into five groups of six mice each (*n* = 6/group). Both males and females (*n* = 3/male or female) were used to minimize sex-related bias. Each group received group 1: Vehicle control (0.9% saline, i.p.), group 2: MB-treated group (dose: 4 mg/kg/day, i.p.), group 3: MO-treated group (dose: 260 mg/kg/day, oral), group 4: Raw MB + MO combination (4 mg/kg/day, i.p + 260 mg/kg/day, oral), or group 5: MOMB-Ag-NPs (nanoformulated MB + MO, 4 mg/kg/day, i.p). Mice were housed under controlled temperature with a 12-h light/dark cycle and free access to food and water. All experimental procedures were approved by the Mansoura University IACUC (MU-ACUC Code: SC.MS.23.07.27) and conducted in accordance with the NIH Guide for the Care and Use of Laboratory Animals.

### Treatments, Dose Selection, and Experimental Design

**Fig. 3:**
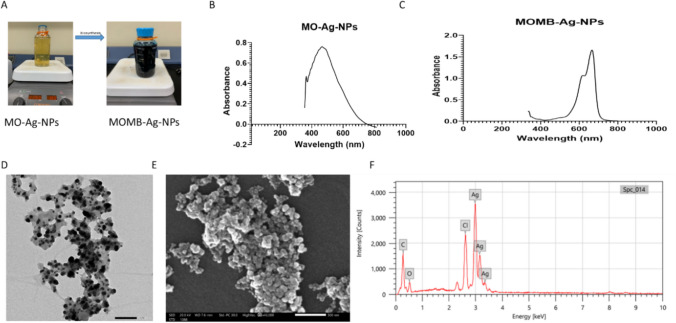
Characterization of MOMB-Ag-NPs. **A** Biosynthesis of MOMB-Ag-NPs, showing the color change from yellow to blue. **B** UV–visible absorption spectra of MO-Ag-NPs, demonstrating a characteristic peak at [specific wavelength, 450 nm]. **C** UV–visible absorption spectra of MOMB-Ag-NPs, demonstrating a characteristic peak at [specific wavelength, 664 nm]. **D** TEM image of MOMB-Ag-NPs, displaying their spherical shape with a scale bar of 100 nm. **E** SEM image with a scale bar of 500 nm. **F** EDX analysis of MOMB-Ag-NPs, illustrating the elemental composition and confirming the presence of silver in the nanoparticles

Methylene blue (MB), *Moringa oleifera* (MO) extract, their combination, and the green-synthesized nanoformulation (MOMB-Ag-NPs) were evaluatedfor their therapeutic efficacy in mitigating Alzheimer’s-like pathology. A pilot dose selection study was first conducted in a separate cohort of P301S mice (*n* = 3/group) to assess tolerability and establish a safe and effective dosing.MB dose selection was guided by previous rodent studies that reported effective outcomes across 2–40 mg/kg/day depending on the administration route [41, 41, 42]. Using allometric scaling, this corresponds to 2–4 mg/kg/day in mice, and a dose of 4 mg/kg/day (i.p.) was adopted in the current study.For MO, previous nutritional and pharmacological studies [25, 43] demonstrated neuroprotective efficacy at 260 mg/kg/day [43], which was selected for oral administration. For the nanoformulation, MOMB-Ag-NPs were administered intraperitoneally at an MB-equivalent dose of 4 mg/kg/day, with nanoparticle mass adjusted to the measured drug loading (DL = 93.5%), corresponding to ~ 4.3 mg/kg. This allowed direct comparison of efficacy and safety between free MB and nanoparticle-encapsulated. In addition to the MB, MO, and MOMB-Ag-NPs groups, a combination group (MB + MO) was initially included. This group received the same MB (4 mg/kg/day, i.p.) and MO (260 mg/kg/day, oral) doses as the single-agent groups. However, all mice (*n* = 6) in this group died between days 28 and 31 of treatment, resulting in early termination of this group. Accordingly, survival analysis was performed to assess the effects of all treatments on survival (Fig. 3). Treatments were delivered once daily, five days per week, for 60 days. This regimen was chosen based on previous long-term therapeutic studies in tauopathy models (~ 50–60 days), balancing sustained exposure with animal welfare [44, 45].

## Behavioral Experiments

### 1- Morris Water Maze

MWM was conducted to assess spatial learning and memory in P301S transgenic mice following the treatment period as described previously [46, 47]. Before starting the treatment, mice were pre-screened to ensure their ability to swim and their general health status. A single swim trial was performed; afterwhich the mice were randomly allocated to the before mentioned five study groups. After treatment, MWM was conducted over five consecutive days, with three trials per day during the training session (day 1 to day 4) then the probe trial (day 5). A probe trial was performed 24 hours after the last training day to evaluate memory retention. An in-house-made water pool was used of 110 cm diameter, and a circular platform with a diameter of 12 cm was positioned at the center of the southeast quadrant (SE quadrant) and submerged 1 cm below the water surface throughout the training sessions. The water was tinted with tasteless, odorless starch. Before the training sessions, the water temperature was set to 24 ± 1°C. Each mouse was gently placed into the water facing the tank wall and allowed to search for the hidden platform for 60 s. The time from water entry to platform arrival was recorded as the latency period. The mice that did not locate the platform within the 60-s timeframe were gently guided to it, where they remained for 30 s to familiarize themselves with the surrounding environment. The mice were subsequently positioned in an adjacent quadrant, and training trials were subsequently conducted in the remaining two quadrants. The training phase continued for four consecutive days, during which the mice were allowed to enter the arena from each quadrant but the SE, where the platform was present. On the fifth day (probe trial), the platform was removed, and the mice were allowed to swim for 60 s in the arena. The recorded videos of training and probe trial days were exported and analysed via the ANYMAZE software.

## 2- Open-Field Test

To test locomotor activity and exploration behavior, each mouse (n = 6 per group) was tested in a 32 cm × 32 cm transparent acrylic box for 5 min under dim light (2.0 lux) [48]. Mice generally prefer to stay near the perimeter of a protective wall rather than explore a central open field. Nonetheless, their ability to forage can encourage the exploration of open areas. The average time spent in the center, latency to initial center entry, number of entries into the center, and total distance travelled were measured and analysed via ANY-maze software.

### Sacrificing and Tissue Processing

Once the behavioral tests were completed, the mice were euthanized with pentobarbital (100 m/kg) and perfused transcardially with ice-cold PBS. Then, the extracted brain was removed from the skull and divided into two hemispheres. One hemisphere was fixed immediately by immersion in 10% (w/v) formaldehyde in 0.1 M saline for immunohistochemistry. The other hemisphere was snap-frozen in liquid nitrogen and stored at − 80 °C for biochemical analyses.

#### Immunohistochemistry and Quantification

Fixed brain samples were dehydrated with increasing concentrations of ethyl alcohol, cleared with xylene and paraffin-embedded. Coronal 5-μm-thick sections were obtained via a rotatory microtome (MicroTec cut 4050, Germany) and mounted on VWR® Superfrost® Plus Microslides for p.Tau and GFAP immunostaining. GFAP immunostaining and quantification were performed as described previously [49] with an anti-GFAP antibody (GFAP (D1F4Q) XP® rabbit mAb #12,389; Cell Signaling Technology, 1:5000). Phosphorylated tau (Ser202, Thr205) (AT8, cat# MN1020, Invitrogen 1:500) was used as described previously [39]. Briefly, after rehydration, the sections were incubated with 0.5% Triton X-100 for permeabilization for 20 min at RT and then incubated with 5% Bovine serum albumin (BSA) as a blocking agent before they were incubated overnight at 4 °C with the primary antibody mixture in 3% BSA. After 3 washes with TBS/0.03% Triton X. the slides were stained with diaminobenzidine (DAB) (mouse and rabbit HRP/DAB (ABC) detection IHC kit, ab64264, Abcam, UK), as illustrated in the datasheet, and the immunoreactivity was visualized as a brown color. Olympus CX41 was used for imaging, and figures were captured for quantification using an Olympus SC100 digital camera. Areas were identified by the counterstains’ nuclear arrangements and nuclei allocation as described earlier in [45]. FiJI was used for quantification. Seven non-overlapping random fields per section from each mouse were captured by an × 40 objective lens and used for quantification of tau reactive signals (AT8) and positive cells per field, which are reported for each group. For GFAP signals the reactive cells were counted and averaged per field for each region and reported in each group as described in [50].

#### ELISA

The hemispheres collected following only PBS transfusion, were finely minced, homogenized in 5 mL of PBS with a glass homogenizer on ice, lysed by freezing (−20 °C)/thawing (RT) 3 times, and centrifuged at 5000 × g for 5 min. The supernatant was collected for assaying. The mouse-specific ELISA kits were used following the manufacturer’s instructions: MAP1LC3B/LC3 (LS-F55215), mouse TNFα (MBS825075), mouse IL-6 (MBS2508516), and mouse superoxide dismutase (SOD) (MBS034842).

#### Western Blotting Analysis

For WB, brain hemisphere tissue was homogenized in ice-cold Radio-Immunoprecipitation Assay(RIPA) lysis buffer (50 mM Tris–HCl, pH 8.0; 150 mM NaCl; 1% NP-40; 0.5% sodium deoxycholate; 0.1% SDS; 1 mM NaF; 1 mM sodium orthovanadate; and protease inhibitor cocktail, Roche). Homogenates were kept on ice and agitated for 30 min at 4 °C, followed by centrifugation at 16,000 × g for 20 min at 4 °C. The supernatant was collected, and protein concentration was determined using the BCA protein assay method following the manufacturer’s datasheet. Equal amounts of total protein (25 μg per sample) were mixed with 6 × Laemmli sample buffer (4% SDS, 10% 2-mercaptoethanol, 20% glycerol, 0.004% bromophenol blue, and 0.125 M Tris–HCl, pH 6.8), heated at 95 °C for 5 min, and briefly centrifuged.Proteins were separated on 10% SDS–polyacrylamide gels using the Mini-PROTEAN® Tetra Cell system (Bio-Rad, USA) at 90 V for stacking and 100–150 V for resolving. After electrophoresis, proteins were electrotransferred onto 0.2 µm PVDF membranes (Sigma-Aldrich) using a Mini Trans-Blot apparatus (Bio-Rad) at 100 V for 45–70 min in transfer buffer (25 mM Tris, 190 mM glycine, and 20% methanol). Membranes were blocked in 5% skimmed milk prepared in TBS-Tween (20 mM Tris, 150 mM NaCl, 0.1% Tween-20, pH 7.5) for 1 h at room temperature and incubated overnight at 4 °C with the following primary antibodies with gentle shaking: phospho-AKT (Cell Signaling Technology, rabbit, 1:2000) and β-actin (Invitrogen, mouse, 1:1000). After washing three times with TBST, membranes were incubated with HRP-conjugated secondary antibodies (Invitrogen, goat anti-rabbit IgG (H + L), 1:3000; rabbit anti-mouse IgG (H + L), 1:3000) for 1 h at room temperature.Protein bands were visualized using enhanced chemiluminescence (ECL, Thermo Scientific) and detected with a Bio-Rad ChemiDoc imaging system. Band intensities were quantified using Image Lab software (Bio-Rad), and β-actin was used as a loading control. The BLUelf Prestained Protein Ladder (GeneDireX, Cat No. PM008-0500) was used as the molecular weight marker. All chemicals and reagents were obtained from Sigma-Aldrich unless otherwise stated. Relative P-AKT expression levels were normalized to β-actin and expressed as fold change compared to the control group.

#### Statistical Analyses

Statistical analyses were conducted with Prism 8.0 (GraphPad), and the normality of the data was assessed via the Shapiro–Wilk test. One-way or repeated-measures ANOVA, followed by the post hoc Tukey test, was used when the data were normally distributed, or a nonparametric Kruskal-Wallistest followed by Dunn’s multiple comparison test was used for the nonnormally distributed data.All data are presented as mean ± SEM, and a p value less than 0.05 was considered significant when *p < 0.05, **p < 0.01, ***p < 0.001, and ****p < 0.0001, except if otherwise mentioned in each figure legend. Densitometric values of Western blot bands were normalized to β-actin and expressed as mean ± SEM.

## Results

### Phytochemical Profiling of *Moringa oleifera* Extract

Phytochemical characterization of MO revealed that the extract possessed a total phenolic content (TPC) of 1374.25 ± 69.08 µg gallic acid equivalents per mL (µg GAE/mL) and a total flavonoid content (TFC) of 1042.86 ± 51.51 µg rutin equivalents per mL (µg RE/mL), indicating a rich abundance of polyphenolic and flavonoid constituents. The antioxidant capacity of the extract was confirmed through radical scavenging assays, with DPPH radical scavenging activity measured at 0.85 mg Trolox equivalents (TE)/mL and ABTS activity at 0.66 mg TE/mL, underscoring its potent antioxidant potential.HPLC–PDA profiling provided further insights into the extract’s composition. Distinct peaks were identified at characteristic retention times, with major constituents including myricetin (rt = 24.9 min, 94.664 µg/mL) and apigenin-7-glycoside (rt = 29.2 min, 55.960 µg/mL), along with phenolic acids such as chlorogenic acid (rt = 13.2 min, 28.432 µg/mL), rosmarinic acid (rt = 30.3 min, 7.280 µg/mL), p-hydroxybenzoic acid (rt = 10.3 min, 7.875 µg/mL), vanillic acid (rt = 16.3 min, 2.929 µg/mL), and gallic acid (rt = 4.1 min, 2.238 µg/mL).

### Ag-Nanoparticle Synthesis and Characterization

#### UV–Visible Spectroscopy

The biosynthesis of MO-Ag-NPs was initially indicated by a visible color change from yellow to light brown (Fig. 3A), confirming the reduction of Ag⁺ ions by MO extract. UV–Vis spectra showed a distinct peak at 450 nm for MO-Ag-NPs (Fig. 3B), verifying successful silver nanoparticle formation. Upon the addition of methylene blue (MB), the solution turned deep blue, and a strong absorption peak at 664 nm was observed, confirming MB functionalization and the formation of MOMB-Ag-NPs (Fig. 3C).

#### Transmission Electron Microscopy (TEM)

TEM analysis revealed nanoparticles with sizes ranging from 10 to 25 nm, displaying predominantly spherical to oval morphologies. The particles appeared well dispersed, with limited aggregation (Fig. 3D).

#### Scanning Electron Microscopy (SEM) and Energy-Dispersive X-ray Spectroscopy (EDX)

SEM images confirmed a spherical morphology with some degree of agglomeration (Fig. 3E). EDX analysis verified the elemental composition, showing the presence of silver (48.3%), oxygen (12.28%), carbon (29.8%), and chlorine (10.35%), confirming successful MO- and MB-mediated stabilization and coating (Fig. 3F).

#### Encapsulation Efficiency and Drug Loading

Nanoparticle synthesis yielded 9.3 mg of dried powder. Drug loading analysis revealed 8.7 mg of encapsulated MB, with 7.2 mg detected in the supernatant as free drug. The encapsulation efficiency was calculated at 54.7%, while the drug loading capacity reached 93.5%, highlighting efficient MB incorporation relative to nanoparticle mass.

### Pilot Animal Study and Dosing Strategy

**Fig. 4:**
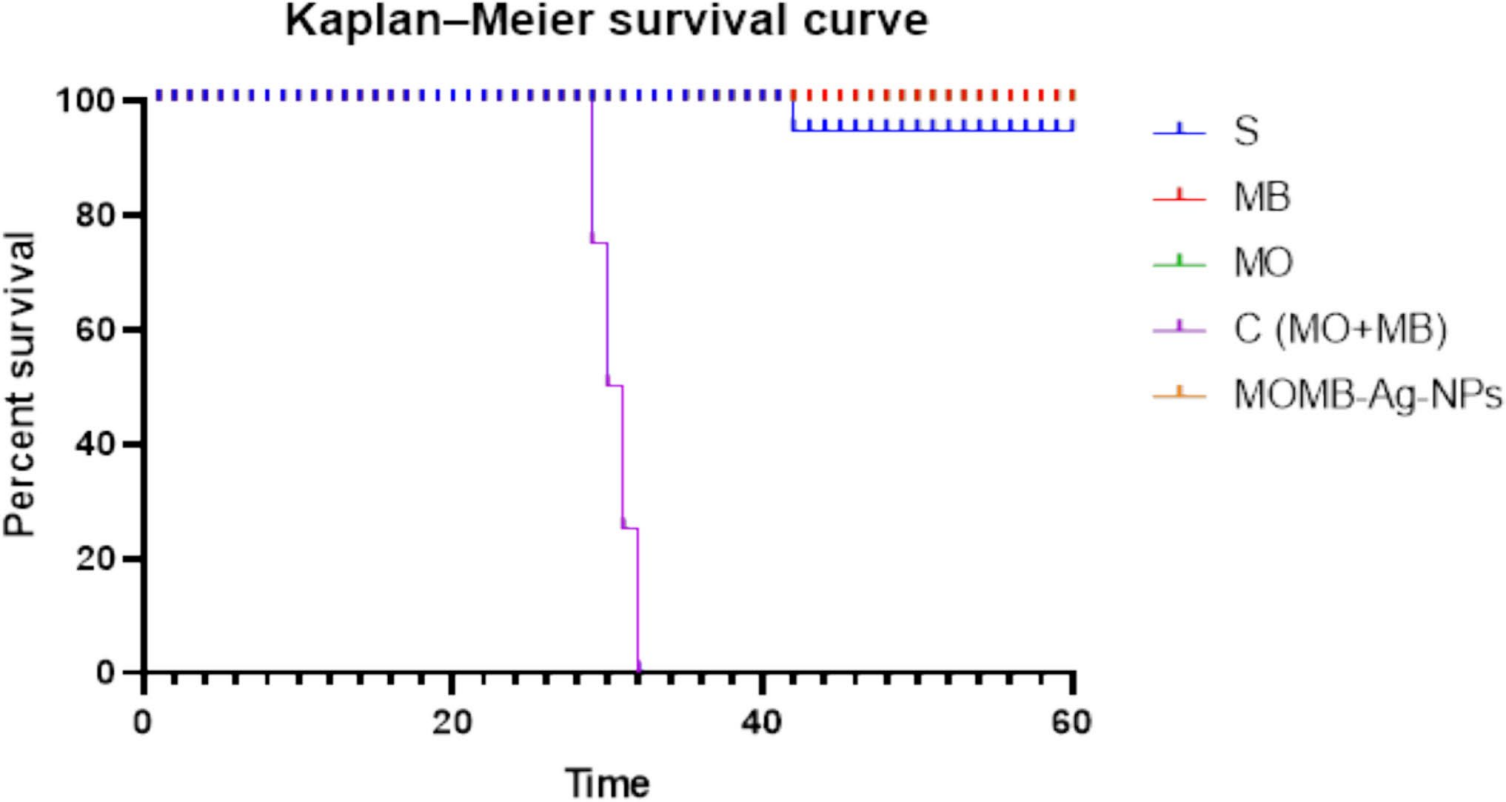
Kaplan–Meier survival curves of P301S mice treated with different interventions. Mice were randomly assigned into five groups (*n* = 6/group; 3 males and 3 females): saline (control), methylene blue (MB, i.p.), *Moringa oleifera* extract (MO, oral), MB + MO (i.p. + oral), and MOMB-Ag-NPs (i.p.). Survival was monitored throughout the 60-day treatment period. The MB + MO combination group showed 100% mortality within ~ 30 days, leading to early termination. The MOMB-Ag-NPs group exhibited complete survival until the end of the study, comparable to MB and MO monotherapies, while the saline group displayed a single spontaneous death on day 43. Note: overlapping curves reflect closely similar values among groups

In the pilot study, route-dependent differences in tolerability were observed. Oral administration of MO extract was well tolerated, while i.p. delivery of MB ensured consistent systemic bioavailability compared to oral dosing. The MB + MO combination, however, resulted in 100% mortality within 30 days (Fig. 4), indicating potential cumulative toxicity when both agents were co-administered despite their individual tolerability.By contrast, the green-synthesized MOMB-Ag-NPs demonstrated improved tolerability, with stable survival across the 60-day observation period and no major adverse events. Based on these findings, the final dosing strategy was selected as follows: MB (4 mg/kg/day, i.p.), MO (260 mg/kg/day, oral), and MOMB-Ag-NPs (MB-equivalent 4 mg/kg/day, i.p.), administered 5 days per week for 60 days. This schedule ensured sustained therapeutic exposure, minimized injection-related stress, and supported animal survival during long-term treatment.

### Survival and Tolerability

The Kaplan–Meier survival analysis (Fig. 4) revealed significant group-dependent differences. The MB + MO combination group exhibited rapid clinical decline, characterized by the loss of spontaneous movement and food intake, leading to 100% mortality within 29–31 days. In contrast, the saline group showed only one spontaneous death without prior clinical symptoms. All animals in the MB, MO, and MOMB-Ag-NP groups survived the full 60-day treatment period. Notably, MB-treated mice displayed mild hypoactivity towards the study endpoint, while MO-treated mice maintained the highest levels of activity and feeding behavior, suggesting better tolerability. Full survival data are summarized in Supplementary Table S1.

## In Vitro Study Results

MO and MB potently inhibited the enzymatic activity of GSK-3β.

To test the efficiency of MO and MB in controlling the activity of GSK-3β, an in vitro enzyme inhibition assay was conducted, and the IC_50_ values were determined (Table 1). MO demonstrated high potency as an inhibitor of GSK-3β, with an IC_50_ value of 9.41 µg/mL. On the other hand, MB was a less potent yet still promising inhibitor of GSK-3β (IC_50_ = 65.77 µg/mL).

Myricetin, the most abundant polyphenolic flavonoid of MO, and MB demonstrated good recognition of the active site GSK-3β along with the formation of multiple interactions with the active site amino acids upon molecular docking.

## In Silico Study Results

**Fig. 5:**
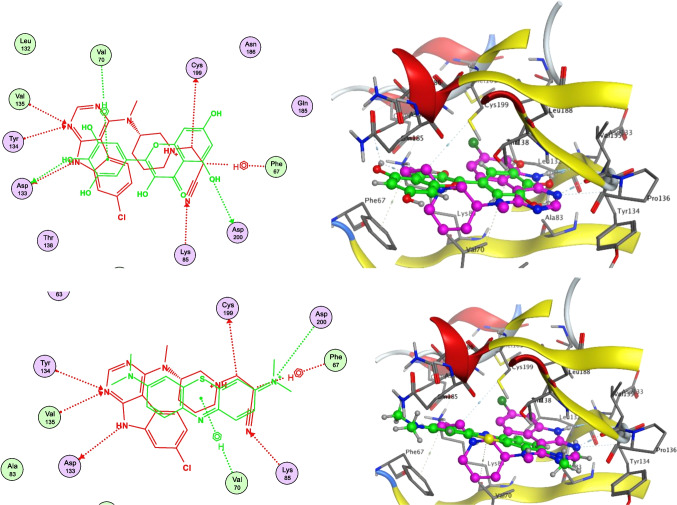
2D and 3D molecular docking results for the active site of GSK-3β (BDP ID: 7B6F): myricetin (green, upper panel); MB (green, lower panel) in comparison to the cocrystallized ligand (PDB ID: SZW) (red in 2D and magenta in 3D)

**Fig. 6:**
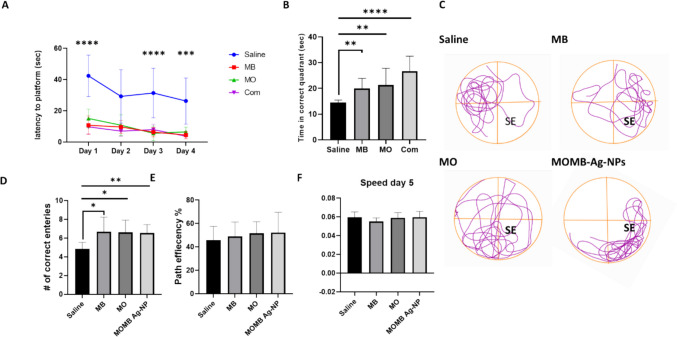
MWM performance improved following MB, MO, and MOMB-Ag-NP combination therapy in P301S mice. **A** Scatter plot showing the escape latency (time taken to reach the hidden platform) over 4 days in the Morris water maze, illustrating decreased latency with treatment. **B** Bar graph representing the time spent in the target quadrant during the probe trial, showing the increased time following treatment. **C** Tracking paths during the probe trial: visual representation of search patterns in the Morris water maze for the saline (control) group, search patterns for the MB treatment group, search patterns for the MO treatment group, and search patterns for the MOMB-Ag-NP treatment group. **D** Bar graph showing the number of entries into the target quadrant, which increased with treatment. **E** Bar graph representing path efficiency in the probe trial, which showed no significant change. (F) Bar graph representing average speed in the probe trial, which showed no improvement. *p < 0.05, **p < 0.01 vs. saline. Values represent the mean ± SEM of each group

A molecular docking study was performed to investigate the molecular mechanism of action of myricetin, and the ability of MB to identify and occupy the active site of its biotarget GSK-3β was demonstrated (Fig. 5). Docking validation was first initiated to affirm the suitability of the selected parameters through redocking of the cocrystallized ligand (PDB ID: SZW), which returned similar poses and interactions to those of the cocrystal complex (RMSD = 0.375 and *S* score = −8.56 kcal/mol). Docking of the polyphenolic flavonoid myricetin resulted in the formation of 3 bonds with the active site amino acids of GSK-3β (Fig. 5, upper panel), *viz.* two hydrogen bonds with Asp133 and Asp200 and a hydrogen‒pi bond with Val70 (*S* score = −6.66 kcal/mol). On the other hand, MB exhibited only two interactions upon docking to GSK-3β (Fig. 6, lower panel), namely, a hydrogen bond with Asp200 and a hydrogen‒pi bond with Val70 (*S* score = −6.11 kcal/mol). This can, in part, explain the observed lower IC_50_ of MB on GSK-3β than that of MO.

### In Vivo Studies

#### Behavioral Experiment Results

To assess whether the administered drugs could mitigate behavioral deficits in P301S mice, the MWM test was employed to evaluate spatial learning and memory performance in P301S tau transgenic mice following treatment. Compared with saline-treated controls, mice receiving MB, MO, or their combined nanoformulation (MOMB-Ag-NPs) exhibited marked improvements in learning performance, as evidenced by a progressive reduction in escape latency across the four training days. Relative to the control group, MB-treated mice showed reductions in latency of 74.6%, 67.0%, 79.8%, and 82.9% on days 1–4, respectively. Similarly, MO treatment led to decreases of 64.4%, 63.1%, 82.1%, and 75.1%, while MOMB-Ag-NPs achieved the most consistent and pronounced reductions of 77.0%, 76.0%, 74.6%, and 85.7% over the same period. These findings indicate enhanced spatial learning and memory acquisition in all treated groups, with the nanoformulation exhibiting the most stable and superior performance throughout the training phase. During the probe trial, all treated mice spent significantly longer durations in the target (SE) quadrant, confirming improved memory retention. The MB group showed a 42.7% increase in time spent in the correct quadrant, while MO treatment produced a 49.5% improvement. Notably, the MOMB-Ag-NP group demonstrated the most substantial enhancement, spending 81.8% more time in the target zone compared with controls. Although all treatments increased the number of correct entries into the platform area, differences among the drug-treated groups were not statistically significant. Path efficiency analysis further supported these behavioral improvements. Compared with saline controls, MB treatment increased navigation efficiency by 16.9%, MO by 10.8%, and MOMB-Ag-NPs by 21.3%, reflecting more accurate and goal-directed swimming toward the platform. Importantly, swimming speed on day 5 showed minimal variation among groups (− 3.4% for MB, + 1.0% for MO, and + 1.7% for MOMB-Ag-NPs versus saline), confirming that the cognitive improvements were independent of locomotor function. Collectively, these data demonstrate that all treatments partially rescued hippocampal-dependent learning and memory deficits in P301S mice, with the combined nanoformulation showing the strongest and most consistent neurobehavioral recovery.

**Fig. 7:**
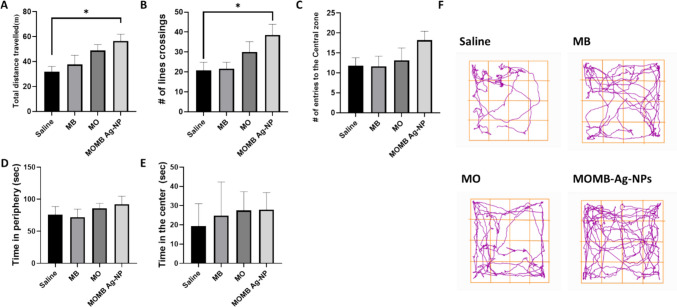
MOMB-Ag-Nps improve locomotor function in P301S mice. **A** Total distance travelled: a bar graph representing the distance (meters) travelled by rodents treated with saline solution (black bar), MB drug (dark gray bar), MO drug (medium gray bar), and MOMB-NP drug (light gray bar) shows the improvement with different treatments compared to saline; MOMB-Ag-NPs *p < 0.05 vs. saline. **B** Number of line crossings, shown by a bar graph, represents a positive effect of the treatment, especially MOMB-Ag-NPs; MOMB-Ag-NPs *p < 0.05 vs. saline. **C** Number of entries into the central zone, shown by a bar graph. **D** Time spent in the periphery: a bar graph represents time spent in the periphery of the open field by rodents. **E** Time spent in the center: duration spent in the center of the open field arena by the different treatment groups as bar graph data. **F** Representative tracking paths: visualization of the movement patterns of the rodents in the saline control group, MB drug treatment group, MO drug treatment group, and MOMB-Ag-NP treatment group during the open field test. Values represent the mean ± SEM of each group

The open field test was performed to assess locomotor activity and anxiety-related behavior in P301S mice following treatment with MB, MO, or their combined nanoformulation (MOMB-Ag-NPs). Although minor differences were observed in total distance traveled among groups, these were not statistically significant, indicating that the treatments did not substantially impair generallocomotor activity. MOMB-Ag-NP-treated mice exhibited the greatest exploratory activity, with a mean traveled distance of 57.9 ± 4.3 m compared with 32.0 ± 3.5 m in saline controls. Similarly, the number of line crossings increased by 68.4% in the combined treatment group relative to saline, reflecting higher locomotor engagement (Fig. 7A, B). Saline-treated P301S mice spent most of their time in the peripheral zone (80.7 ± 5.2 s), indicative of thigmotaxis and elevated anxiety. In contrast, MB, MO, and MOMB-Ag-NP treatments reduced peripheral time by 15.5%, 18.3%, and 26.8%, respectively, accompanied by increased center zone exploration (Fig. 7D). The MOMB-Ag-NP group spent 30.5 ± 2.8 s in the center, representing a 58.9% increase compared with saline controls.

Moreover, the number of entries into the center zone, a measure of reduced anxiety and increased exploratory drive, was significantly elevated in all treated groups. Compared with saline, MB and MO treatments produced increases of 20% and 56.8%, respectively, whereas MOMB-Ag-NPs yielded the most pronounced effect with an 82.4% rise in center entries (Fig. 7C). These findings collectively indicate that while all interventions mitigated anxiety-like behaviors in P301S mice, the combined nanoformulation exerted the most consistent anxiolytic and locomotor-enhancing effects, without altering basal motor performance. Additional behavioral parameters, including time spent in the center (Fig. 7E), are presented to complement the center entries data. Representative movement tracks are shown in Fig. 7F. Percent changes reported here were calculated according to standardized methods suggested for comparative purposes.

#### Astrogliosis is Significantly Reduced Following Treatment of P301S Mice with MB, MO, and MOMB-Ag-NPs

**Fig. 8:**
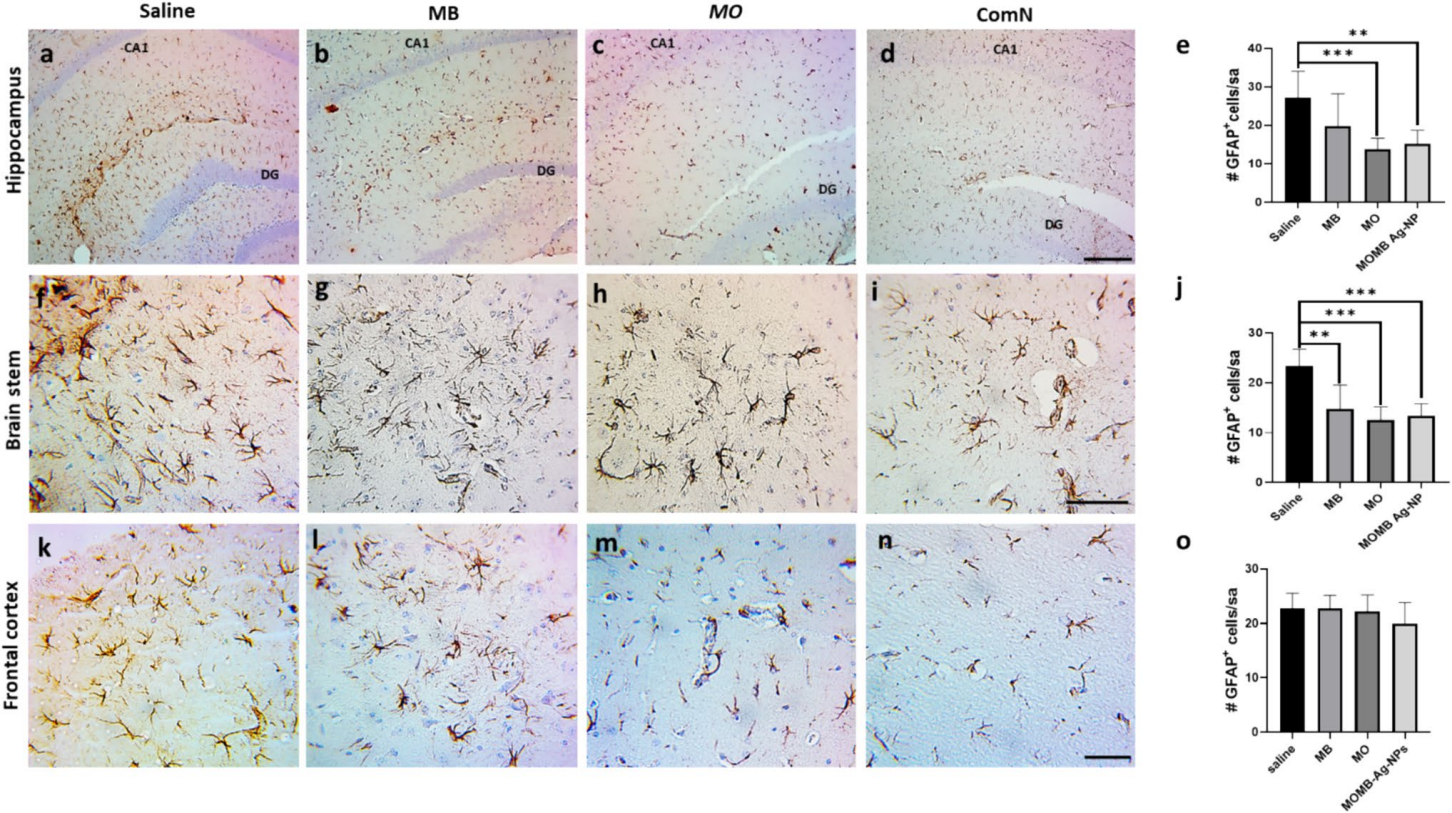
Immunostaining of estrogliosis detected by GFAP + cells is reduced following treatment of P301S mice using MB,MO and MOMB-Ag-NPs Panels a to n immunohistochemistry analysis of GFAP + cells in the hippocampal, brain stem,and frontal cortex region. e, j, o Quantitative analysis of GFAP + cells in the hippocampus, brain stem, and frontal cortex represented by a bar. Compared with the control treatment, MB treatment resulted in a significant reduction in GFAP + cells(**p < 0.01), MO treatment resulted in a significant reduction in GFAP + cells compared with the control group(**p < 0.001), and MOMB-NP treatment resulted in a significant reduction in GFAP + cells compared with the controlgroup (**p < 0.001). Values represent the mean ± SEM of each group

P301S tau transgenic mice are reported to show an extensive accumulation of astrocytes, as demonstrated by GFAP^+^ cells in different brain regions: hippocampus, brain stem, and frontal cortex as demonstrated earlier (Fig. 8a, f, and k). Treatment with MB (Fig. 8b, g, and l), MO extract (Fig. 8c, h, and m), or MOMB-Ag-NPs (Fig. 8d, i, and n) significantly reduced gliosis in the hippocampus and brain stem, with the exception of MB in the hippocampus, which failed to reach statistical significance (Fig. 8e and j). Interestingly, the frontal cortex did not show a significant reduction of gliosis for all groups.

#### Treatment with MB, MO, or MOMB-Ag-A-Nps Does Not Hinder the Accumulation of Phosphorylated Tau in the P310S Brain

**Fig. 9:**
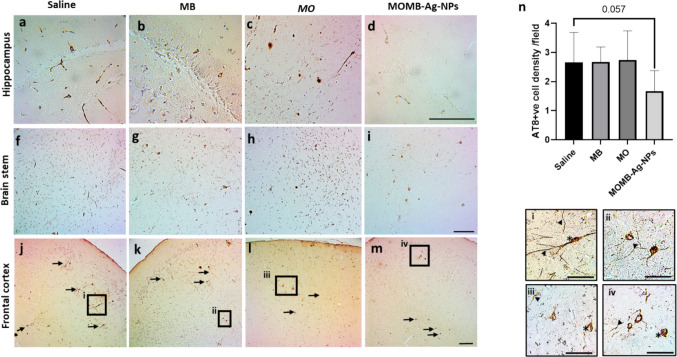
AT8 immunostaining of brain sections. Representative images of AT8 + ve (phosphorylated tau) cells from P301S mice. **a**–**m** Representative figures of AT8 immunostaining of P301S brain sections from each treatment group, at hippocampus, brain stem, and frontal cortex. **i**–**iiii** Zoomed-in images demonstrate the nuclear assembled phosphoTau (asterisks) and the axonal phosphor tau (head arrow). **n** Bar chart demonstrating the quantification of AT8 + cell density per field. The bar graph shows the average density of AT8 + cells per field for the four different treatment groups in the P301S mouse model. Values represent the mean ± SEM of each group

P301S mouse model characterizes with the accumulation of phosphorylated Tau proteins in distinct brain regions as reported earlier, with a distinct flame-like structure that shows both cell body inclusion (asterisks) and axonal structures (arrowheads) (Fig. 9a). Whilenone of the treated groups achieved a statistically significant reduction of p-tau in different brain regions tested, the combination of MOMB-Ag-NPs resulted in a mere reduction in AT8^+^ cell density, with a p value of 0.057 (Fig. 9e), suggesting that MOMB-Ag-NP treatment is a more efficient candidate than the other treatments and can suggest a potential synergistic effect.

##### MOMB-Ag-NP Treatment Most Efficiently Reduced the Inflammatory Biochemical Phenotype, Autophagy, and Antioxidative Effect

**Fig. 10:**
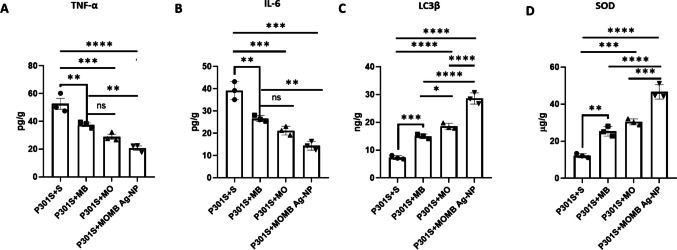
ELISA demonstrating the impact of MB, MO, and MOMB-NPs on TNF-α, IL-6, MAP1LC3β, and SOD levels in the brain tissue of P301S mice. **A** Bar graph showing TNF-α levels. Significant reductions in **B** IL-6 levels were observed in all groups. **C** MAP1LC3β levels. **D** SOD levels. The data are presented as the means ± SEMs. Statistical analysis was conducted via one-way ANOVA followed by Tukey’s post hoc test. Abbreviations: MB, methylene blue; MO, *Moringa oleifera*; MOMB-NPs, methylene blue-*Moringa oleifera* nanoparticles; SEM, standard error of the mean. Statistical significance: *p < 0.05, **p < 0.01, ***p < 0.001, ****p < 0.0001. Values represent the mean ± SEM of each group

In an attempt to identify the potential mechanism of P301S-induced improvements in MWM and reduced gliosis, groups of inflammatory markers were measured by ELISA from the brain homogenates of each group. All treated groups presented reduced levels of inflammatory markers, including TNFα and IL-6. The effect of MOMB-Ag-NPs was more prominent than that of either MB or MO extraction alone, suggesting the potential synergistic effect (Fig. 10A and B). Similarly, MOMB-Ag-NP treatment resulted in the highest levels of LC3β among all the groups, indicating robust autophagy activation (Fig. 10C). MOMB-Ag-NPs notably enhanced SOD activity, indicating activation of endogenous antioxidant defenses that may contribute to protection against oxidative stress (Fig. 10D).

##### Differential Modulation of AKT/GSK-3β Pathway by MB, MO, and MOMB-Ag-NPs Treatments by Western Blot Analysis

**Fig. 11:**
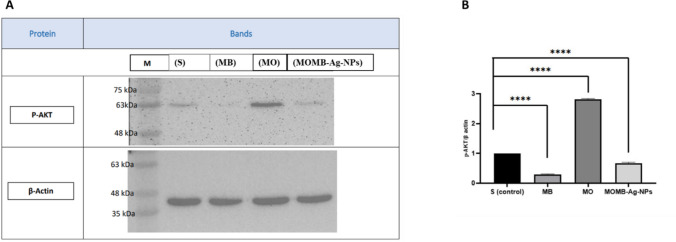
**A** Representative Western blot bands showing p-AKT (upper panel) and β-actin (lower panel) expression levels in saline (S), methylene blue (MB), *Moringa oleifera* extract (MO), and MOMB-Ag-NPs-treated groups. β-actin was used as a loading control. **B** The corresponding bar graph (right) represents the densitometric quantification of p-AKT normalized to β-actin, expressed as fold change relative to the control group. MO treatment markedly increased p-AKT levels, whereas MB and MOMB-Ag-NPs treatments showed reduced expression

Immunoblotting of p-AKT revealed observable variations among treatment groups, which may reflect differential modulation of the upstream AKT/GSK-3β signaling cascades. The MO extract treated group produced a pronouncedelevation of P-AKT ~ 2.83-fold compared to the controlgroups, in consistency with strong suppression of GSK-3β activity as demonstrated in *invitro* experiments. In contrast to the in vitro studies, the MB treated group showed a reduction of the p-AKT signaling, which was interestingly upregulated in the MOMB-Ag-NPs treated group (~ 33% decrease) (Fig. 11). While the exact effect of MB on this signaling is not tested in the current study which may include other regulators and rule players, the current data suggest that introducing the MOMB-Ag-NPs form consistently demonstrates a synergistic effect compared to the solo administration of each drug.

## Discussion

Recent advancements in neurodegenerative research have prompted a paradigm shift in the understanding of AD pathogenesis. Historically dominated by the amyloid-beta hypothesis [51, 52], contemporary evidence underscores a multifactorial etiology involving tauopathies, neuroinflammation, oxidative stress, mitochondrial dysfunction, and impaired proteostasis [1, 53]. This perspective highlights the need for multitarget therapeutic strategies capable of addressing the complex molecular landscape of AD [54, 55]. Clinical translation has been limited by pharmacokinetic challenges, underscoring the need for novel formulations and delivery systems capable of enhancing CNS bioavailability. Nanoparticle-based delivery systems have emerged as promising solutions, offering enhanced brain delivery, reduced systemic toxicity, and sustained release of neuroprotective agents, including phytochemicals and small molecules [56–58]. These developments highlight a therapeutic paradigm integrating multitarget pharmacology with nanomedicine, which guided the present study.Building on this rationale, we propose a novel combinatorial nanotherapeutic approach designed to co-deliver MB and *Moringa oleifera (*MO) extracts via a green-synthesized silver nanoparticle platform (MOMB-Ag-NPs). This system aims to increase brain bioavailability and facilitate simultaneous modulation of multiple pathogenic pathways. In the current study, silver nanoparticles were chosen as the delivery vehicle due to their favorable surface chemistry for phytochemical adsorption, ability to cross the blood–brain barrier, and intrinsic antioxidant and anti-inflammatory properties [30, 59], and MB and MO were tested considering the previously reported effects, including antioxidative and anti-inflammatory effects [25, 28, 60]. Mechanistic alignment across in vitro*, *in silico*,* and in vivo assays further supported this approach. Our in vitro assays demonstrated that MO flavonoids effectively inhibited kinase activity (IC50 = 9.41 µg/mL), consistent with molecular docking results showing strong binding of myricetin to the GSK-3β active site. By contrast, MB exhibited only modest direct GSK-3β inhibition, in line with previous reports indicating that MB primarily affects total tau levels rather than directly modulating GSK-3β [61]. These findings reinforce prior reports of their potential benefits across various neurodegenerative models, including tauopathies and synucleinopathies, and suggest their broader applicability [62].

Phytochemical profiling of the aqueous *Moringa oleifera* (MO) extract revealed a rich abundance of phenolic and flavonoid constituents. For instance, myricetin has been reported to inhibit tau aggregation while exerting potent antioxidant and anti-inflammatory effects, and modulates GSK-3β and MAPK signaling [26, 63, 64], thereby reducing tau hyperphosphorylation and neuroinflammation. These converging effects highlight MO as a potential multitarget neuroprotective candidate. MBwhich is known to inhibit tau aggregation and enhance mitochondrial respiration [60, 65, 66] has gained interest for potential AD therapy; however the later-phase clinical trials revealed dose-dependent toxicity and limited brain bioavailability, constraining its translational potential [17, 41, 61, 67, 68].Therefore, co-encapsulation of MB with MO in a nanocarrier system offers a rational strategy for a possible therapeutic synergy, via improving the pharmacokinetic stability, and potentiate disease-modifying efficacy.

docking analyses further substantiated these functional insights, revealing that myricetin, the predominant flavonoid constituent of MO, forms multiple stabilizing interactions within the GSK-3β active site. This finding suggests a mechanistic basis for the observed enzyme inhibition and points to the potential of these agents to modulate tau phosphorylation pathways critically involved in AD progression.

For an in vivo assessment, different treatment options were administrated to P301S mice models of tauopathy. a model that has been widely used by others and ourselves [37, 39]. Both male and female mice were represented equally in the current study groups. Including both sexes, while still neglected, is vital in the field of neurodegeneration, as sex influences disease risk, progression, and treatment response. For instance, Alzheimer’s is more common in women, while Parkinson’s affects men more frequently, each with distinct clinical patterns [44, 69]. While it is noteworthy that no differences between both sexes were detected in the current assessed parameters in the current study, other non-tested variables could demonstrate differences among both biological sexes. On our hand, the initial attempts to co-administer raw MB and MO extracts resulted in severe systemic toxicity and poor tolerability in vivo and 100% mortalitybetween days 29 and 31, shifting the focus to only the nanoformulation to reduce toxicity and possible side effects. Consequently, the MOMB–Ag–NP formulation was adopted for subsequent experiments as the optimized delivery platform.

Comparable studies have demonstrated the utility of nanoparticle systems for delivering neurotherapeutics. For example, the PEGylated liposomal formulation of MB [70] and Moringa-derived phytochemicals encapsulated in nanocarriers [71]. The development of our green-synthesized nanoformulation represents the central novelty of this study, providing a dual-functional carrier system designed to enhance multitarget therapeutic efficacy against tauopathy. Our strategy not only addresses the pharmacokinetic limitations observed with raw extracts but also leverages the intrinsic properties of AgNPs, such as their ability to cross the BBB and facilitate sustained release, thereby potentially optimizing therapeutic outcomes as demonstrated earlier by others. This approach aligns with emerging trends in nanomedicine, emphasizing multifunctional, biocompatible, and environmentally sustainable nanocarriers for complex neurodegenerative diseases. By employing green-synthesized silver nanoparticles, we capitalize on their biocompatibility and ability to traverse the BBB, which has been demonstrated in multiple recent studies [34, 72].

Comprehensive physicochemical characterization confirmed the robustness of the implemented nano formulation: The initial colorimetric change and the UV–Vis absorbance peaks at 450 nm (Ag-NPs) and 664 nm (MB functionalization) confirm not only the reduction of Ag⁺ ions by *Moringa oleifera* phytochemicals but also the efficient surface functionalization of methylene blue (MB). This dual spectral signature establishes the structural integrity of the nanocomposite and supports its potential for controlled drug delivery. The TEM and SEM analyses demonstrated that the nanoparticles were predominantly spherical, with sizes ranging from 10 to 25 nm and minimal aggregation. Such nanoscale dimensions are particularly advantageous for crossing the BBB and ensuring sustained bioavailability within the diseases related to the central nervous system (CNS) like AD and others. The EDX results further validated the successful integration of organic (carbon, oxygen, chlorine from MB and MO) and inorganic (silver) components, supporting the rationale of employing silver as a stabilizing core. Importantly. from a drug-loading perspective, the nanocarrier demonstrated high encapsulation efficiency (54.7%) and loading capacity (93.5%), highlighting the effectiveness of this biosynthetic approach in incorporating MB relative to nanoparticle mass. This property is of translational importance, as it ensures minimal drug loss and consistent therapeutic delivery. Together, these results demonstrate the novelty of MOMB-Ag-NPs as a multifunctional, stable nanocarrier that integrates phytochemical-mediated biosynthesis with dual-drug delivery to address the multifactorial pathology of Alzheimer’s disease.

Behavioral assessments provided critical evidence of the functional benefits. In MWM, mice treated with MB, MO, or MOMB-Ag-NPs demonstrated significantly reduced escape latency by Day 4, consistent with improved spatial learning. During the probe trial, all treatment groups spent more time in the target quadrant and displayed a higher number of correct entries relative to saline controls, suggesting partial restoration of spatial memory. Although none of the interventions fully normalized memory performance, these results indicate that both monotherapies and the nanoformulation were able to mitigate tauopathy-associated cognitive deficit; still no significant edge was seen in any of the treated groups compared to others. Importantly, swimming speed and path efficiency did not differ across groups, confirming that the observed improvements were driven by cognitive rather than motor performance.

By contrast, the OF test revealed a distinct therapeutic advantage of MOMB-Ag-NPs. Mice that received the MOMB-Ag-NPs exhibited significantly greater total distance traveled and number of line crossings, reflecting improved locomotor activity, while MB and MO monotherapies did not produce similar effects. Exploratory behavior indices, including time spent in the center versus periphery, were not significantly altered, excluding anxiety-related confounds. These findings suggest that the MOMB-Ag-NPs uniquely extended their benefits beyond cognitive rescue to motor domain improvements in P301S mice, a multidimensional effect highly relevant to tauopathy progression. The apparent discrepancy between memory performance in the MWM and locomotor outcomes in the OF likely reflects the distinct behavioral demands of the tasks: whereas MWM performance emphasizes hippocampal-dependent spatial memory with a more aversive nature considering the life-threatening aspects associated with the need to find the safe dry space (ie: platform), the OF more directly captures locomotor capacity and exploratory drive that are impacted by anxiety and stress levels, which may be preferentially restored by sustained dual-target modulation through the nanoparticle system.

At the histopathological level, immunohistochemical analysis of GFAP expression revealed that treatment mitigated astrogliosis in the hippocampusand brainstem apart from the MB treated group in the hippocampus. Interestingly, none of the groups showed an influence on the GFAP expression in the frontal cortex. This can be due to the less abundant astrogliosis in FC as reported earlier that made the effect demonstrated at other parts very much observed here.

However, tau pathology, AT8 immunoreactivity was not hindered in any of our tested groups. While the MOMB-Ag-NP–treated group demonstrated a tendency to reduce ptau accumulation, it still failed to reach statistical significance. This can suggest that the nanoformulation demonstrating more effective results is due to the synergistic effect on tau pathology.

At the molecular level, biochemical analyses substantiated the histological and behavioral findings by revealing a marked attenuation of neuroinflammatory and enhanced endogenous antioxidant defenses markers alongside activation of autophagic clearance pathways. Among all treatment groups, MOMB-Ag-NP administration most efficiently reduced the inflammatory biochemical phenotype, as evidenced by significantly lower TNF-α and IL-6 levels and upregulation of LC3 and SOD compared with both MB and MO monotherapies. This pronounced anti-inflammatory response likely reflects the synergistic interaction between MO’s bioactive flavonoids and MB’s redox-modulating capacity when co-delivered in nanoparticle form, enhancing cellular uptake and bioavailability across brain regions, and can suggest that the behavioral improvement is more dominantly a cause of reduced inflammation rather than Tau load reduction. This aligned with data from the lab suggested that the neuroinflammation is observed in P301S and impacts the early behavioral changes prior to tau aggregation [39], suggesting that the modulation of inflammatory insult in P301S can successfully mitigate the cognitive impairment. The Western blot findings provide mechanistic insight into how each treatment differentially modulates the AKT/GSK-3β signaling axis, a central pathway governing tau phosphorylation and neuronal survival [28, 73–75]. In Alzheimer’s disease and tauopathies, aberrant activation of GSK-3β drives pathological tau hyperphosphorylation and neurofibrillary tangle formation [76], while phosphorylation by AKT at Ser9 acts upstream to inhibit GSK-3β, thereby promoting neuronal protection and synaptic stability.

While the data needs to be further tested by other candidates including GSK-3β itself, in our hand *Moringa oleifera* (MO) extract produced a pronounced increase in p-AKT levels, indicating activation of the AKT pathway and subsequent inhibition of GSK-3β. This effect is consistent with previous evidence that MO’s flavonoids—particularly myricetin and quercetin—enhance PI3K/AKT signaling, downregulate GSK-3β activity, and consequently reduce neurotoxicity and improve memory performance [63]. Such activation of AKT not only promotes neuronal survival but also supports autophagy and antioxidant responses, aligning with the biochemical and behavioral improvements observed in the current work. Conversely, MB markedly suppressed AKT phosphorylation, suggesting reduced inhibitory control over GSK-3β and potential overactivation of this kinase. While the effect of MB on the GSK-3β/AKT pathway is controversial, recent reports suggest that it may not beneficially regulate upstream kinases such as AKT [17, 41, 61, 67, 68]. Moreover, high MB concentrations or prolonged exposure are suggested to paradoxically increase tau oligomerization and phosphorylation through GSK-3β activation and oxidative stress amplification [61]. These findings may explain why MB monotherapy in this study exhibited partial neuroprotection and also can partially explain the contradicting in silico and in vitro data with animal studies in our hand.

Interestingly, MOMB–Ag–NPs mitigated, though did not completely reverse, the MB-induced downregulation of p-AKT. Despite showing p-AKT levels below those of the control group, the formulation partially prevented the suppression observed with MB alone, indicating that MO’s bioactives within the nanocarrier can partially offset MB’s adverse signaling effects. However, the nanoformulation did not fully restore AKT activity or achieve the strong GSK-3β inhibition observed with MO alone; this suggests that the relative proportion of MO to MB in the formulation might be considered for further optimization in future studies. Increasing the MO content or optimizing the drug ratio may enhance the balance between redox regulation and kinase signaling in future formulations, obtaining better results.

Collectively, these findings suggest that while MO exerts a potent inhibitory effect on GSK-3β via AKT activation, MB alone may induce the opposite trend, potentially exacerbating tau phosphorylation. The MOMB-Ag-NPs formulation partially corrected MB’s negative influence but remained below control levels, reflecting the need for formulation optimization. Nonetheless, these results highlight the potential of nanocarrier systems to modulate complex drug interactions in neurodegenerative therapy.

Current FDA-approved drugs for Alzheimer’s disease largely act on symptomatic pathways rather than directly targeting tau pathology or neuroinflammation. Cholinesterase inhibitors (donepezil, rivastigmine, galantamine) increase synaptic acetylcholine by inhibiting acetylcholinesterase or butyrylcholinesterase, yielding short-term cognitive benefits (≈2–3 MMSE points over 6–12 months) without altering tau phosphorylation, neuroinflammation, or disease progression [6, 77–79]. Memantine, a noncompetitive NMDA receptor antagonist, attenuates glutamate-induced excitotoxicity by non-competitively blocking overactivated receptors, thereby protecting neurons from calcium overload and providing modest preservation of daily functioning in moderate-to-severe AD, but similarly fails to modulate tau pathology or proteostatic mechanisms [80]. More recently, anti-amyloid monoclonal antibodies such as aducanumab and lecanemab demonstrated robust amyloid plaque clearance and modest slowing of cognitive decline [6, 81], yet their efficacy is limited to amyloid burden with persistent concerns about ARIA and inconsistent functional outcomes. Methylene blue (MB) and its derivative leuco-methylthioninium bis(hydromethanesulfonate, LMTM) advanced to phase II/III trials as tau aggregation inhibitors and mitochondrial enhancers. Clinical studies reported slowed brain atrophy rates and stabilization of cognitive decline in monotherapy subgroups [82], but phase III outcomes were variable, and higher doses caused tolerability issues such as gastrointestinal, urinary adverse effects, and severe toxicity [27, 41, 61, 68]. Our results with MB alone are in line with previous reports demonstrating its limited efficacy and its potential to enhance tau oligomerization and phosphorylation. *Moringa oleifera* (MO) extracts, tested in preclinical AD models, consistently attenuated oxidative stress (↑SOD, catalase), reduced lipid peroxidation (↓MDA), and yielded partial improvements in learning and memory performance [32, 83, 84]. Additional studies reported decreased Aβ deposition and anti-inflammatory effects through reduced microglial activation [83], but oral administration limited MO bioavailability and CNS penetration, restricting its translational impact. Lastly, our nanoformulation (MOMB-Ag-NPs) demonstrated that it engages multiple intracellular pathways simultaneously. Specifically, it suppressed proinflammatory cytokines (↓TNF-α, ↓IL-6), enhanced endogenous antioxidant defenses (↑SOD), and activated autophagy (↑LC3β), suggesting a potential to modulate the clearance of dysfunctional proteins. The effect that surpasses when MB or MO were administered as monotherapies.

While our data raised interesting results, several limitations must be acknowledged. Although MOMB-Ag-NPs improved cognitive performance and attenuated key pathological features like neuroinflammation, the therapeutic effect was partial, particularly concerning tau pathology, the primary pathological hall mark of the disease. The disease stage at the time of intervention may have limited the potential for full recovery and this opens a potential for future studies that assess the effect of treatment at earlier disease stages. Additionally, the absence of pharmacokinetic, phytochemical, antioxidant and biodistribution profiling of MOMB-Ag-NPs represents a major gap, as the precise bioavailability, blood–brain barrier penetration, and systemic clearance remain undefined. Also, we assessed selected molecular endpoints (tau phosphorylation, enhanced endogenous antioxidant defenses, pro-inflammatory cytokines, and LC3β expression); still more comprehensive mechanistic investigations including specific tau kinases, direct GSK-3β activity measurement and mitochondrial dynamics should be addressed in future studies. The absence of wild-type mice treated with Ag-NPs and MOMB-Ag-NPs as control groups represents another limitation of the present study, that hindered the study of a potential hazardous impact on the wild-type mice.

Furthermore, long-term safety studies addressing nanoparticle accumulation, chronic toxicity, and potential off-target effects are essential to establish translational viability. To bridge the gap toward clinical application, future work should also incorporate pharmacodynamic biomarkers, dose–response profiling, and head-to-head comparisons against existing tau- and amyloid-directed agents. Together, these steps will be essential to validate MOMB-Ag-NPs as a safe and effective disease-modifying strategy for Alzheimer’s disease.

## Conclusion

In summary, our findings demonstrate that MOMB-Ag-NPs provide a multifaceted therapeutic benefit in a P301S tauopathy mouse model, evidenced by improved cognitive test performance reductions in astrogliosis, suppression of pro-inflammatory cytokines, enhancement of antioxidant defenses, and activation of autophagy. These molecular effects translated into improvements in cognitive and motor performance, highlighting the potential of this nanoformulation as a disease-modifying intervention rather than a purely symptomatic therapy. Compared with currently approved FDA drugs, which primarily target cholinergic or glutamatergic transmission, and with previous studies on MB or MO monotherapies, our data support a broader mechanism that addresses multiple pathogenic processes simultaneously. While these results are promising, translation to the clinic will require comprehensive pharmacokinetic profiling, long-term safety studies, and validation in additional Alzheimer’s disease models. Collectively, this work positions MOMB-Ag-NPs as a strong candidate for further development toward innovative therapeutic strategies in Alzheimer’s disease.

## Supplementary Information

Below is the link to the electronic supplementary material.

ESM1 (DOCX.270 KB)

## Data Availability

The dataset supporting the conclusions of this article is included within the article and its additional file.

## Abbreviations

AD: Alzheimer’s disease
S: Saline
MB: Methylene blue
MO: *Moringa oleifera*
MOMB: Nanoformulated combinational therapy of MB and MO
Aβ: Amyloid-beta
NFTs: Neurofibrillary tangles
FDA: U.S. Food and Drug Administration
SOD: Superoxide dismutase
IACUC: Institutional Animal Care and Use Committee
CMP: Center for Microbiology and Phage Therapy
AgNO_3_: Silver nitrate
TEM: Transmission electron microscopy
SEM: Scanning electron microscopy
MWM: Morris water maze
OF: Open-field test
GSK-3β: Glycogen synthase kinase-3 beta
P-AKT: Phosphorylated Akt protein
DPPH: 2,2-Diphenyl-1-picrylhydrazyl
ABTS: 2,2′-Azino-bis(3-ethylbenzothiazoline-6-sulfonic acid)
RIBA: Radio-immunoprecipitation assay buffer
