# Supplementary material for: Synergistic Neuroprotection in Tauopathic Mice via Green-Synthesized Silver Nanoparticles Co-delivering Methylene Blue and Moringa oleifera
Source: Mol Neurobiol. 2025 Dec 28;63(1):331. doi: 10.1007/s12035-025-05534-9 (PMC12743692; doi:10.1007/s12035-025-05534-9)

Supplementary table S1: Full survival data

| Group | Animal ID | Sex | Status | Day of Death | Notes (clinical signs) |
| --- | --- | --- | --- | --- | --- |
| Saline | Sm1 | M | Alive | – | Normal |
| Saline | Sm2 | M | Alive | – | Normal |
| Saline | Sm3 | M | Dead | Day 43 | No obvious signs |
| Saline | Sf1 | F | Alive | – | Normal |
| Saline | Sf2 | F | Alive | – | Normal |
| Saline | Sf3 | F | Alive | – | Normal |
| MB (i.p.) | MBm1 | M | Alive | – | ↓ activity low stage |
| MB (i.p.) | MBm2 | M | Alive | – | Normal |
| MB (i.p.) | MBm3 | M | Alive | – | ↓ activity low stage |
| MB (i.p.) | MBf1 | F | Alive | – | Normal |
| MB (i.p.) | MBf2 | F | Alive | – | ↓ activity low stage |
| MB (i.p.) | MBf3 | F | Alive | – | Normal |
| MO (oral) | MOm1 | M | Alive | – | Active, good appetite |
| MO (oral) | MOm2 | M | Alive | – | Normal |
| MO (oral) | MOm3 | M | Alive | – | Normal |
| MO (oral) | MOf1 | F | Alive | – | Normal |
| MO (oral) | MOf2 | F | Alive | – | Normal |
| MO (oral) | MOf3 | F | Alive | – | Normal |
| MB+MO (oral+i.p.) | Cm1 | M | Dead | Day 28 | Hypoactivity, anorexia |
| MB+MO (oral+i.p.) | Cm2 | M | Dead | Day 29 | Hypoactivity, anorexia |
| MB+MO (oral+i.p.) | Cm3 | M | Dead | Day 31 | Hypoactivity, anorexia |
| MB+MO (oral+i.p.) | Cf1 | F | Dead | Day 28 | Hypoactivity, anorexia |
| MB+MO (oral+i.p.) | Cf2 | F | Dead | Day 29 | Hypoactivity, anorexia |
| MB+MO (oral+i.p.) | Cf3 | F | Dead | Day 31 | Hypoactivity, anorexia |
| MOMB-Ag-NPs (i.p.) | NPm1 | M | Alive | – | Normal |
| MOMB-Ag-NPs (i.p.) | NPm2 | M | Alive | – | Normal |
| MOMB-Ag-NPs (i.p.) | NPm3 | M | Alive | – | Normal |
| MOMB-Ag-NPs (i.p.) | NPf1 | F | Alive | – | Normal |
| MOMB-Ag-NPs (i.p.) | NPf2 | F | Alive | – | Normal |
| MOMB-Ag-NPs (i.p.) | NPf3 | F | Alive | – | Normal |

Supplementary Figure: The full gel and plot images of western blotting analysis for P-AKT


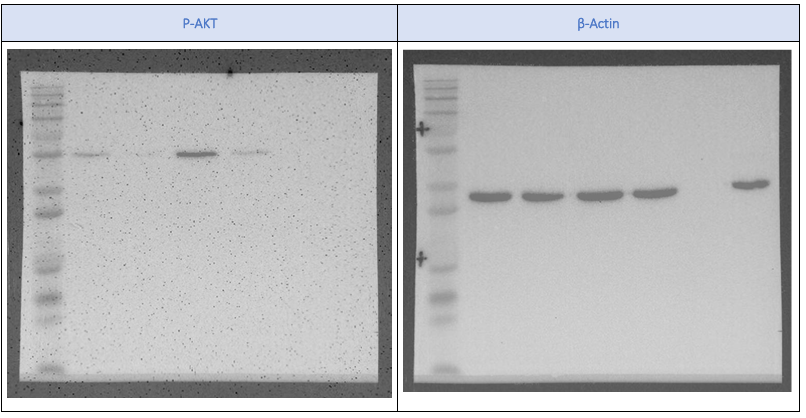

Supplement: Supplementary file 1 — (DOCX.270 KB) [file 12035_2025_5534_MOESM1_ESM.docx]
